# Dr. John Knight

**Published:** 1912-09-28

**Authors:** 


### Obituary.

Dr. John Knight, Medical Officer of Health for Scarborough, has died at Uddingston, near Glasgow. He was only in his thirty-eighth year, and had held the post of medical officer of health at Scarborough for seven years. Dr. Knight was educated in Glasgow, and his appointments included that of resident physician at Glasgow Fever Hospital. He had also been senior assistant to the Glasgow Medical Officer of Health.

